# Assessing the relationship between HIV infection and cervical cancer in Côte d'Ivoire: A case-control study

**DOI:** 10.1186/1471-2334-10-242

**Published:** 2010-08-17

**Authors:** Georgette Adjorlolo-Johnson, Elizabeth R Unger, Edith Boni-Ouattara, Kadidiata Touré-Coulibaly, Chantal Maurice, Suzanne D Vernon, Marcel Sissoko, Alan E Greenberg, Stefan Z Wiktor, Terence L Chorba

**Affiliations:** 1 Projet RETRO-CI, Centers for Disease Control and Prevention, Global HIV/AIDS Program, Abidjan, Côte d'Ivoire; 2 Centre Hospitalier Universitaire de Treichville, Abidjan, Côte d'Ivoire; 3 Centre Hospitalier Universitaire de Yopougon, Abidjan, Côte d'Ivoire; 4 National Center for HIV/AIDS, Viral Hepatitis, STD, and TB Prevention, Centers for Disease Control and Prevention, Atlanta, Georgia, USA; 5 National Center for Emerging and Zoonotic Infectious Diseases, Centers for Disease Control and Prevention, Atlanta, Georgia, USA; 6 National Center for Global Health, Centers for Disease Control and Prevention, Atlanta, Georgia, USA

## Abstract

**Background:**

The association between HIV infection and invasive cervical cancer that has been reported may reflect differential prevalence of human papillomavirus (HPV) infection or uncontrolled confounding. We conducted a case-control study in a West African population to assess the relationship between HIV infection and invasive cervical cancer, taking into account HPV infection and other potential risk factors for cervical cancer.

**Methods:**

Women with invasive cervical cancer (cases) or normal cervical cytology (controls) were recruited in a hospital-based case-control study in Abidjan, Côte d'Ivoire. Odds ratios and 95% confidence intervals (CI) were estimated in logistic regression analyses controlling for important cofactors.

**Results:**

HIV infection was noted in 22/132 (16.7%) cases and 10/120 (8.3%) controls (p = 0.048). High-risk HPV infection was detected in cervical tumor samples from 89.4% of case-participants and in cervical cytology samples in 31.1% of control-participants. In logistic regression analysis, HIV infection was associated with cervical cancer in women with HPV (OR 3.4; 95% CI 1.1-10.8). Among women aged ≤ 40 years, risk factors for cervical cancer were high-risk HPV infection (OR 49.3; 95% CI 8.2-295.7); parity > 2 (OR 7.0; 95% CI 1.9-25.7) and HIV infection (OR 4.5; 95% CI 1.5-13.6). Among women aged > 40 years, high-risk HPV infection (OR 23.5; 95% CI 9.1-60.6) and parity > 2 (OR 5.5; 95% CI 2.3-13.4), but association with HIV infection was not statistically significant.

**Conclusions:**

These data support the hypothesis that HIV infection is a cofactor for cervical cancer in women with HPV infection, and, as in all populations, the need for promoting cervical screening in populations with high prevalence of HIV infection.

## Background

In sub-Saharan Africa, age-standardized incidence of cervical cancer is high, ranging from 29.3 (West Africa) to 42.7 (southern Africa) per 100,000 women [1]. The development of cervical cancer is the result of interaction of systemic and local cofactors that facilitate malignant transformation of cervical cells, with HPV infection as a necessary factor [2]. Based on strength of association with cervical cancer, genital HPVs have been categorized by risk of acting as carcinogens in the development of cervical cancers. High-risk or oncogenic types include HPV types 16, 18, 31, 33, 35, 39, 45, 51, 52, 56, 58, 59, 66, 68, 73 and 82; low-risk types include HPV types 6, 11, 42, 43, 44, 54, 61, 70, 72, 81 [3]. Examples of factors other than HPV that have been suggested as potential modulators of cervical cancer development include age and parity [4,5], cigarette smoking [6], long-term oral contraceptive use [7], and host genetics and immunological factors [8].

The incidence of cervical cancer has been changing at a global level, with increasing incidence in women below 40 years of age [9,10]. This may reflect age-cohort effects and the emergence of more aggressive histologies with a shorter natural history, possibly the result of HPV infection acquired at a younger age or of increased screening/awareness resulting in earlier detection of cervical cancer.

In HIV-infected women, there is an increased risk of HPV infection and squamous intraepithelial lesions (SIL), the precursor of cervical cancer [11,12]. Since 1993, the revised CDC AIDS case definition has included the development of cervical cancer in an HIV-infected person as a sufficient criterion for AIDS, even in the absence of an opportunistic infection [13]. Numerous studies have analyzed the association of HIV infection and cervical cancer [14-16]. Although positive associations between HIV infection and cervical cancer have been demonstrated [15-18], studies evaluating the strength of this association among African women have had differing conclusions [14-16,18,19]. It has been proposed that lack of excess risk of invasive cervical cancer among HIV-infected women in some populations may reflect the competing risk of mortality from other conditions associated with HIV infection [20].

Studies of HIV infection and invasive cervical cancer to date have tended to be limited by lack of information on presence of HPV DNA in cervical samples of study participants, and focused on quantifying the effect of HIV infection relative to other cofactors in the presence of HPV infection. We conducted a case-control study in a West African population to assess the relationship between cervical cancer and HIV infection, taking into account the presence of high-risk HPV infection and other cofactors such as age, parity, and lifetime number of sexual partners. In Côte d'Ivoire, the study setting, the annual incidence of cervical cancer was approximately 24.2 per 100,000 [21] during the study period, and the prevalence of HIV infection among pregnant women attending antenatal clinics, an approximation of the prevalence in the general population, was 10% [22].

## Methods

### Study design and data collection

Over a two-and-a half-year period, all women aged 18-70 years who presented with cervical lesions at the gynecology and oncology services at two tertiary hospitals in Abidjan, Côte d'Ivoire were asked to participate. These university teaching hospitals are the principal referral sites for patients with suspected malignancies in Abidjan. Controls were recruited from consecutive eligible women attending the oncology and general surgery outpatient clinics for post-surgery follow-up evaluation; the principal reasons for their admissions were trauma, abdominal surgery, or breast cancer. None of the patients had evidence of Kaposi sarcoma or non-Hodgkin lymphoma. Eligibility criteria included being aged 18-70 years, having an intact cervix, and having no current or past history of cervical disease. Cases and controls were not individually matched for age, place of residence, or economic status.

All consenting women were interviewed and examined by study personnel, using a standardized questionnaire. Demographic, socioeconomic, reproductive and behavioral data were obtained. Blood samples were collected by venipuncture and a physician performed a complete physical and pelvic examination and collected cervical specimens after visualizing the cervix. For all participants, endocervical and ectocervical cells were collected with a Cytobroom (Cytyc, Malborough, MA) and transferred to PreservCyt™ liquid collection media (Cytyc). For women with suspected cancer, the cervical lesion was biopsied and clinical stage was determined using the 1986 International Federation of Gynecology and Obstetrics (FIGO) architectural staging system [23]. Informed consent for the overall study was obtained from all study participants. All study participants received free clinical evaluation and treatment as indicated by their attending physicians. The study was reviewed and approved by the ethics committee of the Côte d'Ivoire Ministry of Public Health and by the Institutional Review Board of the Centers for Diseases Control and Prevention.

### Definition of case and control

Clinical and pathology data for all participants were reviewed to determine final inclusion into the study. Case-participants were required to have histologically confirmed primary cervical cancer. Advanced disease was defined as FIGO stage III or higher [23]. Control-participants were required to have normal cervical cytology confirmed in the same institutions as those in which the case-participants were patients.

### Laboratory methods

#### HIV serology and CD4 counts

Serum was tested for HIV-1 and HIV-2 antibodies by enzyme-linked immunosorbent assay (ELISA) according to a standardized algorithm [24,25]. Seropositivity and type-specific diagnosis for HIV-1 and HIV-2 infections were determined by reactivity in synthetic peptide-based line immunoassay or combination of monospecific ELISA tests. In Côte d'Ivoire, this HIV testing algorithm was found to have 99% sensitivity and specificity [26]. CD4 lymphocyte phenotyping was performed using a FACScan flow cytometer (Becton Dickinson, San Jose, CA), on EDTA-collected peripheral blood.

#### Cervical samples and HPV DNA detection

Cervical samples and biopsy specimen were sent to the local reference pathology laboratory in Abidjan, and the Centers for Disease Control and Prevention (CDC) laboratory in Atlanta, Georgia. PreservCyt™ samples were shipped at ambient temperature to CDC and used to prepare ThinPrep™ (Hologic, Bedford, MA). Pap smears were read in a diagnostic cytology laboratory using Bethesda 1988 criteria [27]. Residual cellular material was collected by centrifugation, washed in phosphate buffered saline (PBS), re-suspended in 1.5 mL PBS, and stored at -20°C until extraction.

Cervical biopsy tissue specimens were divided in half and transported in formalin. One half was sent to the local pathology laboratory for clinical diagnosis. The other half was transferred to 50% alcohol 1% formalin and shipped at ambient temperature to CDC for processing. At CDC, tissues were trimmed for processing to paraffin blocks, and serial sections were cut from each block. The first and last sections were H&E stained and reviewed to determine research diagnosis. Discrepancies (benign vs. malignant) between clinical and research diagnoses were resolved through consensus after exchange of tissue blocks between the pathology labs. Intervening sections were used for HPV testing by *in situ *hybridization (ISH) and polymerase chain reaction (PCR).

HPV DNA was extracted from 0.5 ml of the stored cervical cytology samples and from two de-paraffinized 5-μ sections of tumor using the QIAmp DNA Mini Kit (Qiagen Inc., Valencia, CA) and concentrated in a Centricon Centrifugal Filter Device YM-100 (Millipore Corporation, Bedford MA) to 25 μL. A 5-μL aliquot was used in the L1 consensus PCR assay (PGMY09 and PGMY11 primer system [28], followed by typing in the Roche line probe assay [29]. Colorimetric ISH for HPV types 6/11, 16, 18, 31, 33 and 35 was performed on formalin-fixed paraffin-embedded sections as previously described [30]. The HPV status for cases was based on combined results from cytology PCR, tumor PCR, and tumor ISH. A case was considered positive when HPV was detected in one or more samples. In the event of a type discrepancy, the result of the tumor PCR was used. The HPV status for controls was based on the PCR result from the cytology sample.

### Statistical analyses

Statistical analyses were performed using PC SAS (version 6.12, SAS Institute, Cary, NC) software. The outcome was histologically confirmed cervical cancer, and the primary risk factor of interest was HIV infection, stratified into two main groups of seroreactivity including three categories of reactivity: HIV-1 positive, HIV-2 positive, and dually-reactive. Because HIV-2 is more indolent than HIV-1 in its effects on the immune system and related pathologies, those who were HIV-1 or dually reactive were combined into one group and association with cervical cancer assessed for the two main groups of seroreactivity, i.e., HIV-1-positive or dually reactive, and HIV-2-positive. Because of the small sample size in a final subgroup analysis by age group, HIV-positive sub-groups were pooled together in one group, and associations with cervical cancer were explored with HIV-negative as the reference. Other potential risk factors considered included age (included as continuous variable), socioeconomic status, number of lifetime sexual partners, parity, (included as categorical variables) and presence of high-risk HPV DNA (HPV types 16, 18, 31, 33, 35, 39, 45, 51, 52, 53, 56, 58, 59, 66, 68, 73, and 82).

In univariate analysis, we compared the characteristics of women with cervical cancer from each of the two hospitals, and then the characteristics of cases and control participants. We then assessed HIV covariates among control participants and the association between HIV infection and presence of high-risk HPV DNA. Comparisons were made using Wilcoxon-rank sum tests for continuous variables, and for categorical variables, Chi Square or Fisher exact test where appropriate [31]. The association of potential risk factors and cervical cancer was assessed by the crude odds ratios and respective 95% confidence intervals.

Multivariate logistic regression analyses were performed to evaluate the independent association between cervical cancer and risk factors that had been identified in univariate analyses. The variables included in logistic regression models were those that were known to be associated with cervical cancer from previous studies and were found to be associated with both cervical cancer and HIV infection in these data. Different models were used in logistic regression analyses. First, we used a model that included age, socioeconomic status, and lifetime number of sexual partners, parity, and presence of high-risk HPV DNA and HIV infection to assess the independent effect of HIV infection taking into account all other cofactors. Second, because HPV sequences are found in more than 99% of cervical cancers worldwide [32], we assumed that our HPV-negative cervical cancers were false negatives, and excluded HPV infection from the model in subsequent analyses assessing effect of HIV infection on cervical cancer. Third, the analysis was restricted to cases and control participants with high-risk HPV infection, and separate logistic regression analyses were done according to age group, accounting for the differential age distribution of case and control participants, and the strong link between age and HIV infection. Fourth, we compared HIV-positive and HIV-negative women with cervical cancer with respect to age at diagnosis and clinical stage to assess whether HIV infection was a predictor of advanced stage and frequency of HPV detection.

## Results

From April 1997 to October 1999, 141 women with putative invasive cervical cancer seen at the hospitals during the study period were asked to participate. Of these, one woman declined and eight were excluded because of lack of histological confirmation, leaving 132 case participants with histologically confirmed cervical cancer. Of these 132, 114 had been recruited from the first hospital and 18 from the second hospital. No significant differences were observed between these two groups with respect to demographics, economic status, the number of lifetime sexual partners, parity or HIV infection status. A total of 135 consenting women were enrolled as potential controls, representing approximately 70% of those eligible from those recruited. Of these 135 women, 120 with normal cervical cytology results were retained as controls; 14 women with cervical dysplasia (including Atypical Squamous Cells of Undetermined Significance (ASCUS) and SIL) and one with missing cytology results were excluded.

### Characteristics of the study participants

Table 1 shows the demographic characteristics and distribution of risk factors for cervical cancer among cases and control participants. Case participants were slightly older than controls and had higher percentages with low economic status and with no formal education. However, cases and controls had similar percentages who reported having had first sex before 16 years and having had > 6 sexual partners during their lifetime. Cigarette smoking was rare both among cases and among control participants. Twenty-two (16.7%) women with cervical cancer and 10 (8.3%) control women were HIV-infected (p = 0.048). None of the women with HIV infection were on antiretroviral therapy (ART); during the study period, ART was not routinely available in Côte d'Ivoire.

**Table 1:** Characteristics of case and control participants

Characteristics	Cases (n = 132)	Controls (n = 120)	p-value
Mean age (range)	46.7 (24-69) %	43.5 (23-69) %	0.02
Age > 40 years	68.2	56.7	0.059
Married	70.5	60.0	0.08
Low economic status	53.8	32.5	0.0001
No formal education	65.2	40.0	0.0001
Cigarette smoking	0	2.5	0.1
First sex < 16 years	37.9	31.7	0.3
Lifetime # of sex partners > 6	15.1	23.3	0.2
History of Pap smear	7.7	16.4	0.04
Parity > 2	87.1	62.5	0.0001
High-risk HPV	89.4	31.1	0.0001
HIV-positive	16.7	8.3	0.048
HIV-1-positive*	12.9	7.5	0.16
HIV-2-positive	3.8	0.8	0.1

* Includes 16 HIV-1-positive and only 1 dually-reactive among women with cervical cancer, and 8 HIV-1-positive and only 1 dually-reactive among control women

The five most prevalent types of HPV found in cervical cancer specimens were: HPV16 (45%), HPV18 (21%), HPV45 (10%), HPV35 (8%), and HPV31 (3%). Other high-risk HPV types found were: 33, 39, 51, 52, 53, 56, 58, 59, 66, and 68. The HPV types in cases and control specimens were similar. However, the prevalence of high-risk HPV infection was higher among women with cervical cancer than among controls (89.4 vs. 31.1; p < 0.0001).

Table 2 presents risk factors (covariates) of HIV infection among control participants. The percentage of women with low economic status, or women with no formal education did not significantly differ between HIV-positive and HIV-negative controls. However, a higher percentage of HIV-positive controls were from low socioeconomic status. HIV infection was significantly associated with age < 40 years, parity > 2, lifetime number of sex partners > 6, and having high-risk HPV infection.

**Table 2:** Covariates of HIV infection among control participants

Variables	HIV-Positive (n = 10)	HIV-Negatives (n = 110)	p-value
	%	%	
Age group < 40 yrs	70.00	40.91	0.0578
No education	40.00	40.00	1.0000
Low economic status	60.00	28.18	0.0671
Cigarette smoking	10.00	1.82	0.2315
Age first sex < = 16 years	30.00	31.82	1.0000
Lifetime # of sex partners > 6	50.00	20.91	0.0521
History of Pap smear	10.00	17.98	0.4871
Parity > 2	30.00	65.45	0.0393
High-risk HPV*	55.56	28.87	0.1329
Low-risk HPV	0.00	9.28	0.4352

*HPV DNA results were available for 9 HIV-positive control participants

High-risk HPV types were detected in all (100%) of the HIV-positive women with cervical cancer and in 98/110 (89%) of HIV-negative women with cervical cancer (p = 0.1). In multivariate analysis, only HIV infection was associated with HPV infection among controls (OR = 3.0; 95% CI 0.7-13.5). A stronger association was observed among younger women, but the number of samples were small and the difference was not statistical significant (OR = 6.8; 95% CI 0.5-98.0).

### Risk factors for cervical cancer

Table 3 shows risk factors associated with cervical cancer in univariate and logistic regression analyses. In univariate analyses, women with cervical cancer were more likely to be either HIV-1-infected or HIV-2-infected than were control women; however, these differences were not statistically significant. In logistic regression analysis, when controlling for age, low socioeconomic status, and lifetime number of sex partners > 6, the variables that remained associated with cervical cancer were having high-risk HPV infection (OR 23.0; 95% CI 10.5-50.2), parity > 2 (OR 5.5; 95% CI 2.3-13.4), and low economic status (OR 2.4; 95% CI 1.2-4.9). However, when high-risk HPV infection was removed from the model, HIV-1 infection or dual HIV infection was associated with cervical cancer (OR 3.4; 95% CI 1.4-8.3). Although HIV-2 infection was also associated with having cervical cancer, the association did not achieve statistical significance, perhaps due to the small number of HIV-2-positive women (OR 3.9; 95% CI 0.4-35.6). The positive association of HIV infection and cervical cancer remained unchanged when the analysis was restricted to case and control participants with evidence of high-risk HPV DNA (OR 3.4; 95% CI 1.1-10.8).

**Table 3:** Risk factors for cervical cancer in univariate and logistic regression analyses

Risk Factors	cOR (95% CI)	aOR*(95% CI)	aOR†(95% CI)
Age group > 40 years	1.6 (1-1.7)	1.6 (0.7-3.6)	1.0 (1.0-1.04)
Low economic status	2.6 (1.3-5.3)	2.4 (1.2-4.9)	2.2 (1.3-3.8)
Lifetime # of sex partners > 6	0.6 (0.3-1.1)	0.8 (0.3-2.0)	0.8 (0.4-1.7)
Parity > 2	4.1 (2.0-8.4)	5.5 (2.3-13.4)	3.6 (1.7-7.4)
High-Risk HPV	21.2 (10.4-42.9)	23.0 (10.5-50.2)	NA
HIV-positive	2.2 (1.0-4.9)	1.8 (0.6-5.3)	3.4 (1.4-8.3)
HIV-1-positive	1.8 (0.8-4.3)	1.8 (0.6-5.6)	3.3 (1.2-8.7)
HIV-2-positive	4.7 (0.5-40.7)	0.9 (0.1-8.5)	3.9 (0.4-35.6)

cOR: Crude odds ratio in univariate analysis

aOR*: Adjusted odds ratio in logistic regression analysis controlling for high-risk HPV

aOR†: Adjusted odds ratio in logistic regression analysis uncontrolled for high-risk HPV

Of women aged ≤ 40 years, those with cervical cancer were similar to controls with respect to economic status and number of lifetime sexual partners. Table 4 shows the risk factors for cervical cancer in separate logistic regression analyses by age group. The risk factors associated with having cervical cancer were having high-risk HPV infection and parity > 2, both in women aged ≤ 40 years and in older women. However, HIV infection was associated with cervical cancer only in younger women (OR 4.5; 95% CI 1.5-13.6). There was no confounding of the relationship by age, socioeconomic status, or lifetime number of sexual partners. However, a potential interaction between high-risk HPV and HIV infection could not be assessed.

**Table 4:** Risk factors for cervical cancer by age group in logistic regression analysis

Risk Factors	Age < 40 years			Age > 40 years		
	**Cases ** (n = 42) %	**Controls ** (n = 52) %	OR* (95% CI)	**Cases** (n = 90) %	**Controls** (n = 68) %	OR* (95% CI)

Low economic status	40.5	25.0	2.6 (0.7-9.6)	54.4	26.5	2.1 (0.8-5.3)
Lifetime sex partners > 6	35.7	32.7	2.0 (0.5-7.1)	5.6	16.2	0.2 (0.04-1.1)
Parity > 2	71.4	42.3	7.0 (1.9-25.7)	94.4	77.9	5.1 (1.2-21.9)
High-risk HPV	95.2	43.2	49.3 (8.2-295.7)	86.7	22.6	23.5 (9.1-60.6)
HIV-positive	35.7	13.5	4.5 (1.5-13.6)	7.8	4.6	2.0 (0.4-9.3)

* Adjusted odds ratio in logistic regression analysis

Table 5 shows the characteristics of HIV-positive and HIV-negative women with cervical cancer. All HIV-positive case-participants had evidence of high-risk HPV infection. Among HIV-positive women, cervical cancer was detected at a younger age (median age 39 years (range 24-64)) than among HIV-negative women (median age 47 years (range 28-69)). Nevertheless, a similar percentage (50% each) of HIV-positive and HIV-negative women had advanced stage cervical cancer (Clinical Stage III or IV) at diagnosis. Only 18% of HIV-positive women with cervical cancer had CD4 counts below 200 cells/μl.

**Table 5:** Characteristics of HIV-positive and HIV-negative women with cervical cancer

Characteristics	HIV-Positive (N = 22)	HIV-Negative (N = 110)
		
Median Age (Range)	39 (24-64)	47 (28-69)
Clinical Stage III or IV	50%	50%
Median CD4 Counts (Range)	376 (82-1600)	833 (131-2248)
CD4 Counts < 200 cells/μl	18%	NA

Although HIV-positive women with cervical cancer had lower median CD4 counts than did HIV-positive controls, the difference was not statistically significant (376 cells/μl vs. 572 cells/μl: p = 0.6).

## Discussion

### HIV infection

The results in this case-control study are consistent with established associations of cervical cancer with oncogenic types of HPV infection and with a potential association of cervical cancer with high parity both for older and younger women [5,18,33]. An association between HIV infection and cervical cancer was also noted especially in women aged < 40 years, and this too is consistent with published observations [34].

### HIV-2 infection

Relatively unique to West Africa is the issue of HIV-2 infection. In this study, a higher prevalence of HIV-2 was observed among participants with cervical cancer than among controls; however, the small numbers of women with HIV-2 infection in the absence of HIV-1 infection limited the power of the study to achieve a statistically significant result. An association of cervical carcinoma and HIV-2 infection has been previously noted in studies conducted in West Africa [16,35]. Although HIV-2 infection is associated with an increased likelihood of cervical HPV clearance compared with that for HIV-1 infection, after accounting for CD4 cell count, women infected with HIV-2 do not appear to be appreciably more likely to clear cervical HPV infection than those infected with HIV-1 [36].

### Age and stage

Young age and advanced stage of cervical cancer in HIV-infected women in this study were consistent with published findings of rapidly progressive or more advanced forms of cervical cancer at a young age [37]. A possible explanation is that HIV infection may facilitate the progression of cervical HPV lesions to cancer in young women, and an inverse relationship of high-risk HPV prevalence and age has been described [38]. However, among both the HIV-positive and HIV-negative patients in this study, those with cervical cancer had advanced clinical stages at the time of detection. This may reflect low prevalence of screening, and in a setting where preventive services are not developed, women may seek treatment only when they have advanced disease. There was no evidence that the relationship observed between HIV infection and cervical cancer reflected higher risk behavior among younger women, as numbers of sexual partners was adjusted for in the multivariate analysis.

Although HIV infection itself has been reported as a risk factor for cervical cancer [5,18,37], it may be that infection with high-risk types of HPV facilitates becoming infected with HIV if exposed [39]. A higher prevalence of HPV infection in HIV-positive than in HIV-negative women has been described [38]. Although the association of cervical cancer with HIV infection was observed only among the younger group of participants in our data, it may be that the lower prevalence of HIV infection among older participants resulted in an insufficient number of HIV-infected participants to detect a statistically significant difference in HIV-infection between cases and controls.

### Other biologic considerations

Previous findings support the biological plausibility of an association between HIV infection and development of cervical cancer [40]. It is possible that mechanisms associated with HIV infection *per se *in the presence of HPV infection, as well as the degree of HIV-induced immune suppression, are both contributory to development of invasive cervical cancer. HIV is present in vaginal cells, and there is amplification of HPV expression by HIV *tat *protein, which may explain a direct biologic interaction between HIV and HPV [41], with HIV up-regulating the persistence of HPV, leading to subsequent development of SIL and invasive cervical cancer [42]. Degree of immune suppression associated with HIV infection is correlated with the risk of developing two other AIDS defining cancers, Kaposi sarcoma and non-Hodgkin lymphoma [43]. Although there appears to be a correlation between degree of HIV-induced immune suppression and development of cervical neoplasia [40], in the study presented here, HIV-infected women with cervical cancer did not have evidence of severe immune suppression (CD4 counts < 200 cells/μl).

## Limitations

In interpreting the results of this study, a few limitations should be noted. First, controls who were recruited in the oncology and surgical outpatient clinics may not have been representative of the general population with respect to the probability of having HIV infection; however, if the pathology that resulted in their seeking care at the clinic had been related to HIV infection, the measure of effect would be biased towards the null hypothesis of no association between HIV infection and cervical cancer. Second, women who were recruited in university hospitals may not have been representative of women with cervical cancer in the general population in terms of probability of having HIV infection; to adjust for this potential bias (Berkson bias), control participants were selected from oncology and surgical outpatient clinics of the same institutions from which the case patients were selected. Third, control-participants were on average younger and had a higher socioeconomic status than did case-participants; however, these differences were addressed in multivariate analysis, and therefore were unlikely to affect validity of results. Fourth, the few cervical cancer specimens in which high-risk HPV DNA was not identified were probably false negatives, but their number was sufficiently small that their inclusion in the case group would not have appreciably altered the conclusions of this study.

## Conclusions

The data presented here using confirmatory HIV testing, HPV DNA testing, and histologically confirmed biopsies support associations of cervical cancer with high-risk HPV infection and with parity observed in previous studies. In addition, strong association of HIV infection and cervical cancer was observed among young women, with a high prevalence of high-risk HPV DNA in women with HIV infection. In this study population, few participants had ever had a Pap smear, and cervical cancer was observed at a young age among women with HIV infection in the absence of extreme immunosuppression. The findings overall add support to the association of invasive cervical cancer among HIV-infected persons, an association on which the recommendation for annual cervical cytology screening in persons with HIV infection is based.

## Competing interests

The authors declare that they have no competing interests.

## Authors' contributions

GA-J conceived and designed the study, performed data analysis, and drafted the manuscript; ERU and SDV contributed to study design, and completed cytology and HPV testing; EB-O, KT-C, and CM contributed to study design, and performed clinical exams and HIV testing; AEG and SZW provided scientific support for design and contributed substantially to manuscript writing; TC contributed substantially to revising parts of the study and to manuscript writing. All authors read and approved the manuscript.

## Disclaimer

The findings and conclusions in this manuscript are those of the authors and do not necessarily represent the views of the Centers for Disease Control and Prevention.

## Pre-publication history

The pre-publication history for this paper can be accessed here:

http://www.biomedcentral.com/1471-2334/10/242/prepub
